# Subacromial resurfacing with fascia lata autograft for irreparable cuff tears: JEO 2021 expert opinion short report & surgical technique with video

**DOI:** 10.1186/s40634-021-00359-6

**Published:** 2021-08-17

**Authors:** Nuno Gomes, Helder Fonte, Sara Santos, Duarte Sousa

**Affiliations:** 1 Hospital da Luz Arrábida, Porto, Portugal; 2 Hospital das Forças Armadas, Porto, Portugal; 3 Centro Hospitalar Póvoa de Varzim - Vila do Conde, Póvoa de Varzim, Portugal; 4 Centro Hospitalar do Porto, Porto, Portugal; 5 Centro Hospitalar de S. João, Porto, Portugal

**Keywords:** Subacromial resurfacing, BAR, Bursal acromial reconstruction, Irreparable cuff tears, Fascia lata autograft

## Abstract

Massive and irreparable rotator cuff tears remain a major challenge for orthopaedic surgeons and several surgical options have been accepted for different patterns of irreparable cuff tears. However, the high-cost and technical complexity of some surgeries and the inconsistent clinical results of others, leave room for the search for surgical options that can offer a good balance between technical simplicity, a lower cost and an acceptable clinical result.

The rationale behind some available techniques includes lowering the humeral head, which enables a more efficient balance of the force couples around the shoulder during rehabilitation. The interpositional arthroplasty with a biodegradable balloon, the Superior Capsule Reconstruction and, more recently, subacromial resurfacing with a dermal allograft have been presented as valid options to address that purpose.

This technical note presents Subacromial Resurfacing with fascia lata as an option for the treatment of irreparable rotator cuff tears in the elderly population. Its low-cost and reasonable technical demand, along with the need for further elucidation of the ideal indications and technical optimization of other available techniques may open up room for the success of this option.

**Supplementary Information:**

The online version contains supplementary material available at 10.1186/s40634-021-00359-6.

## Introduction

Massive and irreparable rotator cuff tears remain a major challenge for orthopaedic surgeons. As of today, several surgical options have been accepted for different patterns of irreparable cuff tears. If a conservative approach fails, partial repairs with or without interposition grafts, tendon transfers, long biceps tenotomy, arthroplasties, superior capsular reconstruction (SCR) or a subacromial biodegradable spacer have been presented as successful alternatives [17]. However, the high-cost and technical complexity of some surgeries and the inconsistent clinical results of others leave room for the search for surgical options that can offer a good balance between technical simplicity, a lower cost and an acceptable clinical result.

Conservative treatments for these tears are commonly associated with poor clinical outcomes due to an imbalance of the force couples that result in unstable kinematics of the glenohumeral joint. This leaves the remaining shoulder function sustained by a significantly increased compensatory deltoid force [11]. The rationale behind using a balloon biodegradable spacer takes this into account, as it offers an easy solution to lower the humeral head, enabling a more efficient balance of those force couples during rehabilitation. Also, it allows a smooth gliding of the joint with reduced painful friction between the humeral head and the acromial undersurface, easing the restoration of range of motion and effective function of the deltoid muscle post-operatively [12, 13, 17, 20].

SCR has emerged as a promising technique in this setting. Current evidence suggests that restoring the integrity of the superior capsule can help improve stability, and therefore functional outcomes, by lowering and centering the humeral head [9]. But the appropriate thickness of the graft and its role as a spacer has been widely discussed, suggesting that this may be an important effect of the surgical construct for improved results, considering that the healing rate of the graft may be low despite successful clinical results [2]. The original description of the graft was a tensor fascia lata autograft, which was folded to achieve a thickness of around 8 mm [7]. It has been demonstrated that a graft that size, which exceeds the typical human dermal allograft thickness, is biomechanically superior to a 4-mm graft [2]. Bearing this in mind, Makovicka et al. [5] proposed a technique with promising early results, in which an acellular human dermal allograft is used to perform a superior capsular reconstruction and the remainder is used to resurface the undersurface of the acromion, fixed through acromial holes, doubling the thickness of the graft and its spacer function.

Ravenscroft et al. [14] has recently published an alternative to the previous technique, in which the subacromial resurfacing alone is performed with a dermal allograft, also with promising early results in an elderly population. He named it Bursal Acromial Reconstruction (BAR), assuming the similarity to one of the effects of Superior Capsular Reconstruction (SCR), which involves lowering the humeral head due to the spacer effect of the graft.

It was based on the same rationale that the authors have developed a surgical technique that addresses the same population, with modifications that may increase acceptance for widespread usage. The technique presented consists of a low-cost subacromial resurfacing with fascia lata autograft, harvested through a minimally invasive approach, without the need for routine resection of the lateral end of the clavicle or perforation of the acromion.

## Surgical technique (with video illustration)

The patient is positioned in the beach chair position (author’s preference) but the procedure can also be performed in lateral decubitus. The standard setting for a shoulder arthroscopy is prepared and draping is set in a fashion that allows an easy approach to the lateral aspect of the ipsilateral thigh.



**Additional file 1.**



Indication for the procedure is confirmed after a diagnostic shoulder arthroscopy.

### Fascia lata autograft (FLA) harvesting

**Fig. 1:**
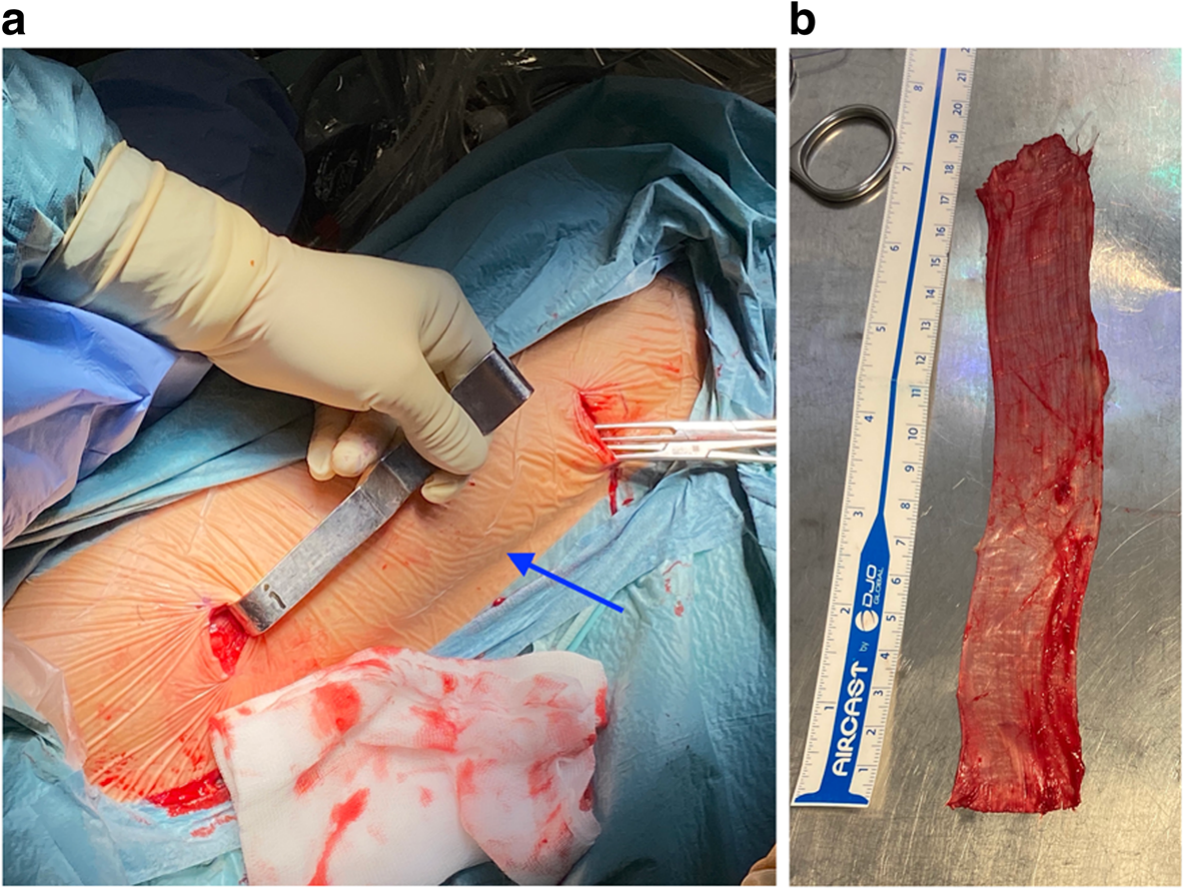
**a** Harvesting of the fascia lata autograft from a right thigh through 2 incisions, approximately 4 cm anterior to the crease of the lateral intermuscular septum (arrow): one 15 cm distal to the anterior superior iliac spine and the other 10 cm proximal to the lateral femoral epicondyle. **b** Autograft should be roughly 20 cm × 3 cm in size

The FLA is harvested through two horizontal (transverse) 2–3 cm long skin incisions, approximately 4 cm anterior to the lateral intermuscular septum, often identifiable as a crease on slim patients: one 15 cm distal to the anterior superior iliac spine and the other 10 cm proximal to the lateral femoral epicondyle (Fig. 1a). Using a Cobb dissector, all adhesions are released superficial and deep to the incised fascia through the minimally invasive approach as described by other authors [1]. Longitudinal parallel cuts of the fascia are completed with long scissors in order to produce a graft roughly 20 cm × 3 cm in size (Fig. 1b). The graft is excised through the proximal skin incision by pushing it through from the distal incision with an Allis forceps.

After skin closure, a compressive dressing is immediately applied to the thigh to avoid hematoma formation during the remaining surgery and replaced by a compressive stocking at the end of the procedure.

### Arthroscopic preparation

A thorough subacromial bursectomy through a standard posterior viewing portal is performed, followed by a minimal acromioplasty in order to provide a flat bleeding bed for graft application. Soft tissues debridement must be complete enough to enable proper identification of the anterolateral and posterolateral corners of the acromion, as well as the limits of the acromioclavicular joint.

In the presence of exuberant exostoses on the humeral surface, a tuberoplasty [3, 18] on the humerus is performed with the reshaping of the greater tuberosity to create a smooth, congruent acromiohumeral articulation.

### Graft preparation

**Fig. 2:**
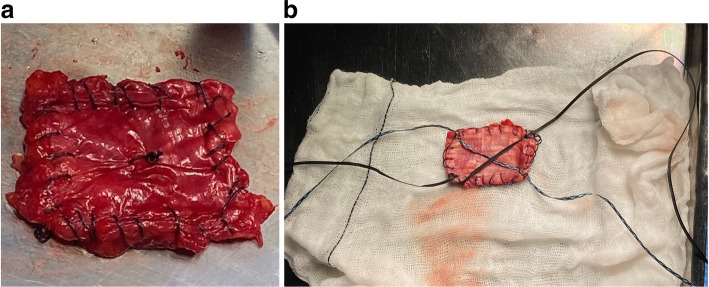
**a** Final prepared graft should be approximately 4 × 3 cm in size, with variable thickness. Thickness of the graft is greater when harvested more proximally. **b** A crossed suture tape configuration with “lasso-loop” knots at each corner is created by passing two BroadBand Tapes (ZimmerBiomet, Warsaw, IN, USA) from corner to corner sequentially, using a Quattro Suture Passer (ZimmerBiomet). The suture tapes are therefore locked to the graft corners, keeping it spread wide open when held under tension

The graft is prepared on a side table by removing all remaining fatty tissue, folding it four to five times and stitching the edges. The prepared graft should be approximately 4 × 3 cm in size (Fig. 2a), with variable thickness. More proximal harvesting will result in thicker grafts but care should be taken to avoid important damage to the tensor fascia lata.

A crossed suture tape configuration with “lasso-loop” knots [4] at each corner is created by passing two UHMWPE (Ultra High Molecular Weight Polyethylene) BroadBand Tapes (ZimmerBiomet, Warsaw, IN, USA) from corner to corner sequentially, using a Quattro Suture Passer (ZimmerBiomet). The suture tapes are therefore locked to the graft corners, keeping it spread wide open when held under tension (Figs. 2b and 5a), in a fashion that offers a good purchase of the graft, preventing it from sliding during delivery and subacromial fixation, as demonstrated before with the usage of a dermal graft [14].

### Graft insertion and fixation

**Fig. 3:**
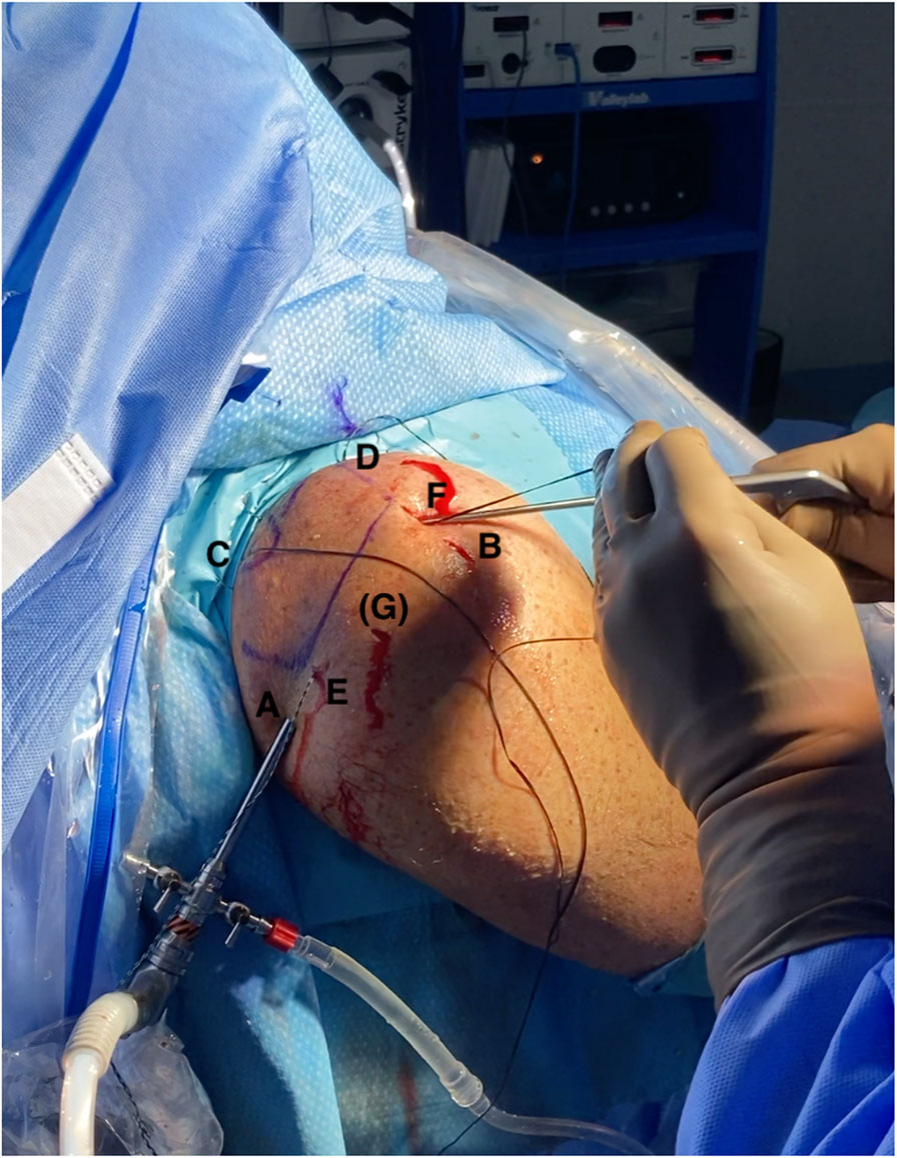
Cannula-free arthroscopic portals and 4 milimetric incisions for tapes passage. Right shoulder, patient in beach-chair position. A: standard posterior viewing portal; B: lateral portal for working and graft passage; C: incision for posteromedial tape / Neviasier; D: incision for anteromedial tape; E: incision for posterolateral tape; F: incision for anterolateral tape; (G): accessory lateral portal may be necessary for better viewing when handling anterior tapes subacromially

The next steps of the procedure are performed with the arthroscope from a posterior viewing portal and a standard lateral working portal used for the previous subacromial preparation, with no cannulas. The scope can be switched to the lateral portal according to the needs of the procedure (Fig. 3).

A needle is used outside-in to locate the millimetric portals posterior and anterior to the acromioclavicular joint (ACJ). The posterior one is close to the classic Neviasier portal and none of them should harm the ACJ.

A suture retriever is introduced through the former to bring outside the tape from the posteromedial corner of the graft, transported to the subacromial space by a grasper. With the graft still outside the body, the tape from the anteromedial corner is also transported to the subacromial space by a grasper, making sure both suture tapes are not tangled and no soft tissues are interposed. A half-pipe cannula may be used for this purpose and the lateral portal opened wider to about 15 mm to allow later transportation of the graft. The tape from the anteromedial corner of the graft is retrieved through the portal anterior to the ACJ and the graft is ready to be shuttled to the subacromial space.

The medial tapes are pulled from the medial portals and the graft, folded in half and held with a grasper, is inserted into the subacromial space. Once the graft is in place, the tapes from the other two lateral corners of the graft need to be passed through the skin, close to the anterolateral and posterolateral corners of the acromion. A needle in an outside-in technique is used again to identify the proper location of these skin incisions, which, like the medial ones, should be beyond the limits of the graft – i.e. separated by a length that is more than the respective size of the graft - to allow an effective pull in opposite directions. This enables proper tensioning of the graft with no sagging.

**Fig. 4:**
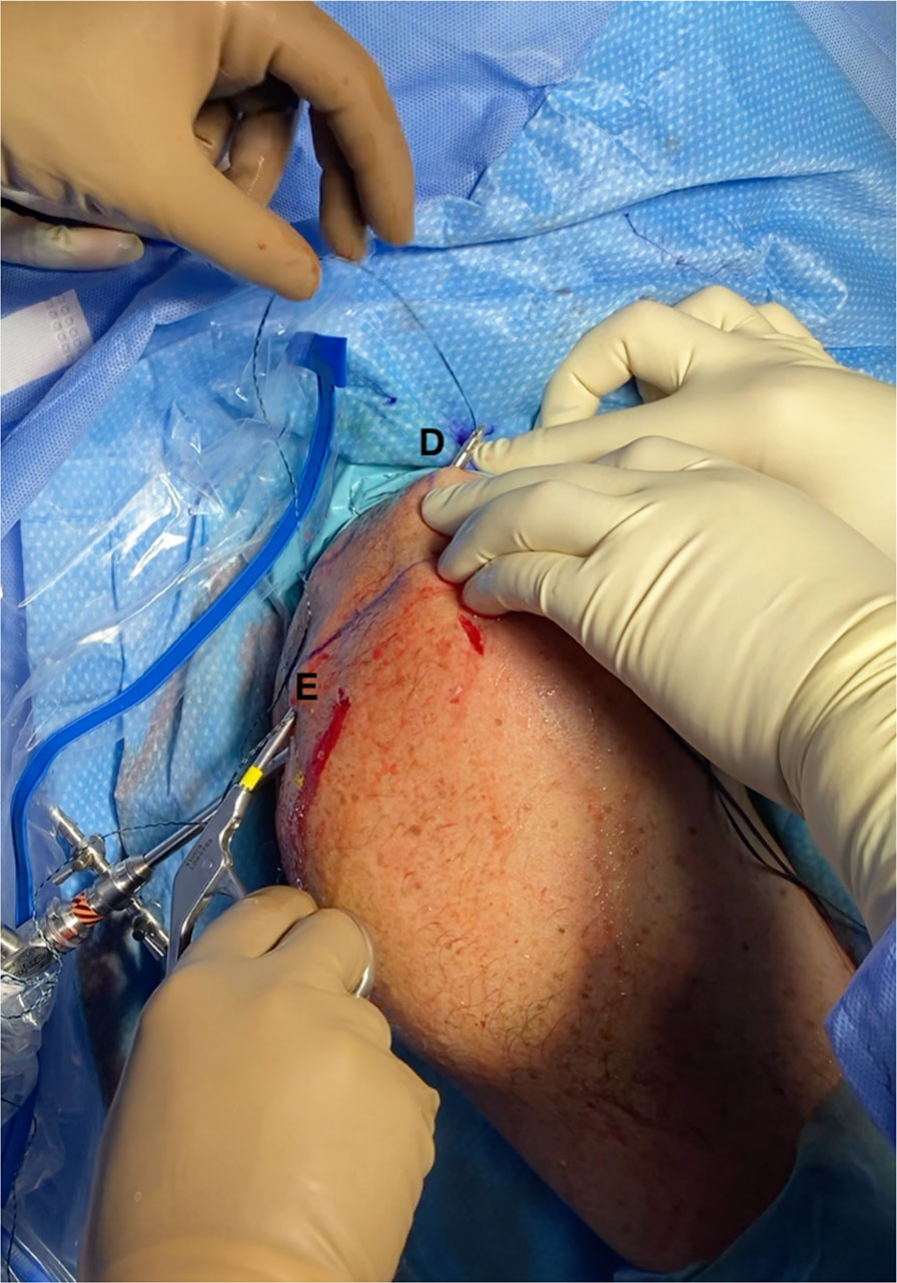
A suture retriever is introduced from the posterolateral acromial incision (E), then slid along the superior surface of the acromion and out through the opposite anteromedial incision (D). The tape tail from this portal is retrieved back through the (E) incision, followed by the same step with the other tape (between incisions F and C). The corresponding tape tails of the same colour are then wrapped and tied around the superior surface of the acromion, in a crossed fashion and using a knot pusher

**Fig. 5:**
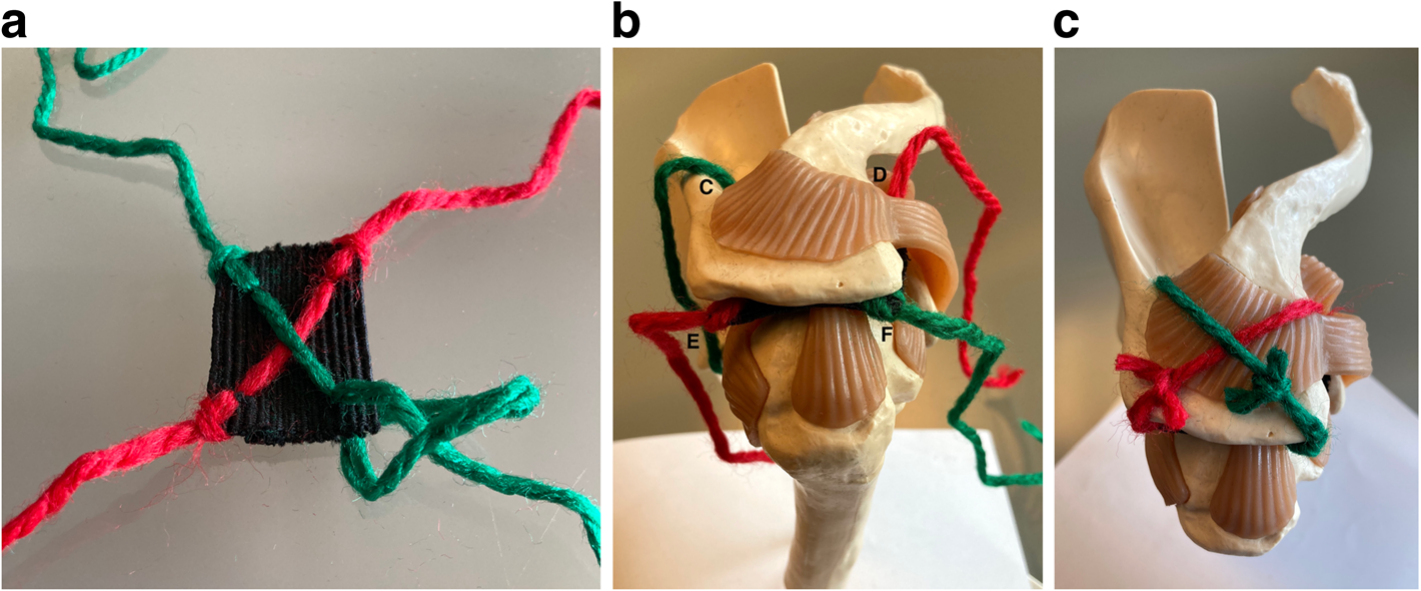
Schematic display of the construct in a model. **a** Crossed suture tape configuration with “lasso-loop” knots at each corner of the graft. **b** Graft in a subacromial position and suture limbs exteriorized through their respective skin incisions. **c** Finalized graft positioning after tying over the acromion

Once the 4 suture tapes are passed outside through the skin, the corresponding tails of each BroadBand Tape (ZimmerBiomet, Warsaw, IN, USA), with the same colour, are retrieved for tying. As described for the BAR technique [14], a suture retriever is introduced from the posterolateral acromial portal, then slid along the superior surface of the acromion and out through the opposite anteromedial portal (Fig. 4). The tape tail from this portal is retrieved back through the posterolateral acromial portal, followed by the same step with the other tape. The corresponding tape tails of the same colour are then wrapped and tied around the superior surface of the acromion in a crossed fashion, using a knot pusher and ensuring the graft is properly positioned (Fig. 5b and c).

## Post-operative care and rehabilitation

The patient is discharged within 24 h and the usage of a compressive stocking is advised for the first 4 weeks after surgery, avoiding excessive demand of the involved lower limb.

Considering the absence of long follow-ups for this procedure, the shoulder rehabilitation programme was defined using clinical reasoning, parallel with other established procedures and observations of early post-operative outcomes.

The early post-op period focuses on passive mobility below the pain threshold to avoid stiffness and wearing an arm sling for 3 weeks. Increasing passive supported movements are implemented according to tolerance. From week 4 onwards, gradual resistance is added, until a dedicated programme for deltoid strengthening is implemented around the 6th week.

## Discussion

Fenlin et al. [3] described, in 2002, the tuberoplasty with the creation of an acromiohumeral articulation as a treatment option for massive irreparable rotator cuff tears, followed by other authors [18] that have named it a reversed subacromial decompression. The humeral gliding against the acromion would become more congruent and therefore less painful, without the risk of superior migration of the humerus that follows an acromioplasty. However, with no recentering of the head, the unbalanced force couples of the shoulder would be maintained, with ineffective recruiting of the deltoid.

Assuming that the interposition effect is the cornerstone of the presented technique, a comparison to the subacromial balloon is inevitable. Recent reports on this procedure show inconsistent results. Ruiz-Ibán et al. [15], in a prospective study with a 2-year follow-up, presented unsatisfactory results in 9 out of 15 patients (60%) after the implantation of a biodegradable spacer, 5 of which requiring reconversion to a reverse arthroplasty. Prat et al. [13] showed unsatisfactory improvement in patients treated with an interpositional balloon with a mean follow-up of 14.4 months and Stewart et al. [21], in a systematic review, reported favorable patient-reported outcomes with limited short-term follow-up. Despite inherent methodological limitations and patient heterogeneity between studies that may impair the ability to fully characterize the long-term efficacy of this technique, it seems that the positive effect of the balloon declines early over time. This is probably due to the biological degradation of the device - within 12 months - and the uncertainty of how long the spacer will remain inflated [16]. Our technique has the potential to offer enough longevity to prevent those early failures. Besides, the risk of implant migration, allergy to the balloon polymer (polylactide-co-epsilon-caprolactone) and its cost are not negligible.

Makovicka et al. [5] and Ravenscroft et al. [14] have recently proposed using a dermal allograft for resurfacing of the undersurface of the acromion for the treatment of selected irreparable cuff tears. The former proposed doing it simultaneously with a SCR and the remainder of the allograft, while the latter proposed doing it alone, based on the assumption that a subacromial interposition arthroplasty, acting as a spacer, is effective in both recentering the humeral head and in avoiding painful friction with the acromion. Following the same rationale, we propose a similar procedure but instead using a fascia lata autograft, harvested by a minimally invasive approach and providing a permanent spacer effect.

Despite the aggression to the harvesting site, which has been shown to be well tolerated, with functional scores of 91% and 94% of the healthy thigh’s score at 6 and 18 months respectively [1], there are several advantages in using this option instead. There is neither the need for acromial perforation as Makovicka et al. [5] proposed with potential risk of fracture, especially in the event of a previous acromioplasty, nor for excision of the lateral end of the clavicle as proposed by Ravenscroft et al. [14], since only 2 suture tapes are used without aggression to the ACJ (without an additional 2 sutures through the long medial and lateral sides of the graft). Furthermore, using an autograft is more biocompatible, with lower risks of immune rejection or failure, is a low-cost solution and can be thicker than a dermal allograft, offering a more effective spacer effect. This ability to decrease superior translation of the humeral head has been shown to be more effective with a thicker autograft in patients undergoing SCR, with higher overall success rates [2, 5].

SCR, an option that has gained popularity in the past few years, improves shoulder function by a tenodesis effect and acting as a spacer, which reverses superior humeral head migration [6, 8, 10, 19]. SCR is theorized to work by successfully rebalancing force coupling between cuff structures, improving both the compression and depressor effects of the rotator cuff and joint capsule. It is indicated in patients with a compensated shoulder function at younger ages, where the infraspinatus is still functional, with no or minimal osteoarthritis. However, indications continue to evolve since it is a relatively new technique. Although more than 15,000 SCRs have been done worldwide, there remains a paucity of outcome data and one must be vigilant to not allow enthusiasm to overtake critical evaluation [22].

BAR has similar indications, with the main difference being the older age of the patient [14]. However, its theoretical biomechanical mechanism differs from that of SCR, since there is no tenodesis effect between the glenoid and the humeral head. Nevertheless, the spacer effect of the graft also plays a role in SCR and should not be underestimated. That same spacer effect with a thicker graft further enhances the appeal of the Subacromial Reconstruction with fascia lata presented in this report. It is technically less demanding than the SCR, faster to perform and less costly (an SCR may need up to 7 anchors, besides the dermal allograft if that is the graft of choice). In spite of not directly addressing the function of the shoulder, since there is no attempt to reconstruct the active function of the cuff as muscle transfers or reverse arthroplasty do, it seeks to offer a secondary functional improvement by diminishing pain and recovering impaired shoulder biomechanics in the patient with rotator cuff deficiency.

No long-term results of this technique are available today to ascertain its advantage over others. Still, the non-negligible rate of complications associated with other demanding surgeries, along with inconsistent success rates, opens up room to consider other alternatives. The Subacromial Resurfacing with fascia lata is an option that may fill a gap among the solutions for these patients and does not “burn any bridges” since any eventual subsequent surgery will be performed in a joint with no major alteration of the native anatomy. This technique may offer an additional advantage in revision cases since it does not require good bone stock in the humeral head, which is a concern if several anchors from previous surgeries are present.

## Conclusion

This technical note presents Subacromial Resurfacing with fascia lata as an option for the treatment of irreparable rotator cuff tears in the elderly population. Its low-cost and reasonable technical demand, along with the need for further elucidation of the ideal indications and technical optimization of other available techniques may open up room for the success of this option.

## Abbreviations

ACJ: Acromioclavicular Joint
BAR: Bursal Acromial Reconstruction
SCR: Superior Capsule Reconstruction
FLA: Fascia Lata Autograft
